# Design and evaluation of an incoherent feed-forward loop for an arsenic biosensor based on standard iGEM parts

**DOI:** 10.1093/synbio/ysx006

**Published:** 2017-12-08

**Authors:** Federico Barone, Francisco Dorr, Luciano E Marasco, Sebastián Mildiner, Inés L Patop, Santiago Sosa, Lucas G Vattino, Federico A Vignale, Edgar Altszyler, Benjamin Basanta, Nicolás Carlotto, Javier Gasulla, Manuel Giménez, Alicia Grande, Nicolás Nieto Moreno, Hernán R Bonomi, Alejandro D Nadra

**Affiliations:** Universidad de Buenos Aires, Facultad de Ciencias Exactas y Naturales, Departamento de Química Biológica, iGEM 2013 Buenos Aires Team, Buenos Aires, Argentina

**Keywords:** I1-FFL, iGEM, arsenic, biosensor, mathematical modeling

## Abstract

The diversity and flexibility of life offers a wide variety of molecules and systems useful for biosensing. A biosensor device should be robust, specific and reliable. Inorganic arsenic is a highly toxic water contaminant with worldwide distribution that poses a threat to public health. With the goal of developing an arsenic biosensor, we designed an incoherent feed-forward loop (I-FFL) genetic circuit to correlate its output pulse with the input signal in a relatively time-independent manner. The system was conceived exclusively based on the available BioBricks in the iGEM Registry of Standard Biological Parts. The expected behavior *in silico* was achieved; upon arsenic addition, the system generates a short-delayed reporter protein pulse that is dose dependent to the contaminant levels. This work is an example of the power and variety of the iGEM Registry of Standard Biological Parts, which can be reused in different sophisticated system designs like I-FFLs. Besides the scientific results, one of the main impacts of this synthetic biology project is the influence it had on team’s members training and career choices which are summarized at the end of this article.

## 1. Introduction

Arsenic contamination in drinking water currently affects hundreds of millions of people worldwide. The most affected countries include Argentina, Bangladesh, Chile, China, India, Pakistan, Mexico, and the USA [World Health Organization (WHO): http://www.who.int/mediacentre/factsheets/fs372/en/]. In Argentina, the population potentially exposed to arsenic-contaminated water is ∼4 million people (1). Arsenic is a systemic poison, and its chronic ingestion can lead to a wide range of health problems, which are collectively called arsenicosis or chronic arsenic poisoning. The effects include skin lesions; skin, lung and bladder cancer and gastrointestinal and pulmonary diseases (2). Because of the spatial variability of arsenic in groundwater aquifers, there are often some arsenic-free sources even in highly contaminated areas (3).

There are several methods to detect and measure arsenic concentration in water, including field-test kits, but they usually require sophisticated equipment, trained technicians, hazardous residues disposal and/or expensive procedures (4,5). Biosensors are proposed as an alternative to overcome these disadvantages (6). Several approaches have been adopted to construct sensitive arsenic biosensors, including both whole-cell and cell-free devices (4,5,7). Taking into account that a portable device to easily and reliably measure arsenic levels would benefit millions of people, many iGEM teams have focused on the development of arsenic biosensors in the past. All of them were based on the ArsR protein, which is the repressor of the arsenic resistance operon present in many bacteria (6). The University of Edinburgh iGEM 2006 team (http://2006.igem.org/University_of_Edinburgh_2006) designed a system that senses arsenic combining the repression of urease and the induction of LacZ to produce a change in pH as the output. After incubation, a pH reduction could be detected with a regular pH electrode. In 2010 and 2011, this system was used by the Debrecen iGEM team to analyze water samples from Hungary by adding bromothymol blue and visual inspection of the color change (http://2010.igem.org/Team: Debrecen-Hungary/arsenic; http://2011.igem.org/Team: Debrecen_Hungary). In 2012, three different iGEM teams developed arsenic biosensors as well. The Ivy-Tech South Bend team (http://2012.igem.org/Team: IvyTech-South_Bend) upregulated the expression of repressor ArsR to increase the threshold for the activation of the reporter protein RFP; however, they did not report on a successful detection range. The UANL Mty-Mexico 2012 team (http://2012.igem.org/Team: UANL_Mty-Mexico/Project) coupled firefly luciferase to the *ars* promoter (*P_ars_*) and modeled their expression at different extracellular arsenic concentrations. Using a different readout, the Cornell 2012 team (http://2012.igem.org/Team: Cornell) also used *P_ars_* to regulate the metal reduction pathway (MtrB) of *Shewanella oneidensis* to produce an electrical current as the output, which could be used in a bioelectrochemical reactor to measure arsenic levels in real time. Then, in the 2013 iGEM competition, we designed and developed an arsenic sensor circuit based on an incoherent feed-forward loop (I-FFL; Team Buenos Aires: http://2013.igem.org/Team: Buenos_Aires/_biological_design; explained below). More recently, the Bielefeld-CeBiTec 2015 iGEM team (http://2015.igem.org/Team: Bielefeld-CeBiTec/Project/CFPS) designed and characterized a cell-free strip-test biosensor to simultaneously measure different heavy metals, including arsenic. Their molecular design for the arsenic biosensor entails the expression of ArsR coupled to sfGFP from freeze-dried *Escherichia coli* extracts immobilized in a paper strip. After the incubation time, using a smartphone camera and an app, the fluorescent signal can be measured with the appropriate pair of emission and excitation filters. However, the design still requires further optimization to improve both detection limits and background noise. Finally, as part of the ‘Herb taster’ project, Hsi-Taiwan 2016 team (http://2016.igem.org/Team: HSiTAIWAN) designed a multiplex biosensor device that can detect whether the levels of both aflatoxin and four heavy metals, including arsenic, are above the regulation limits in Chinese traditional medicines. The arsenic measurement was achieved using a GFP generator under the *P_ars_* promoter (Bba_K1106004), a Standard Biological Part contributed to the registry by our team.

To the extent of our knowledge, the many arsenic biosensors developed to date are still not suitable for on-site and low-cost arsenic detection by a non-trained user. Some of them need analytical laboratory equipment, while others are extremely dependent on the assay conditions (e.g. time and temperature). We set the goal to develop an arsenic biosensor that fulfills the requirements of being reliable, easy to use, portable and inexpensive to produce a real impact, being able to reach low-income populations. Thus, during the 2013 iGEM competition, we worked both on the assembly of an arsenic biosensor, implementing a proof-of-concept device and field experiments, and on a theoretical model to help us for future experimental improvements. In this report, we will focus on the building and results of our model and also on the experimental validation of the parts involved in the biosensor.

**Figure 1.**
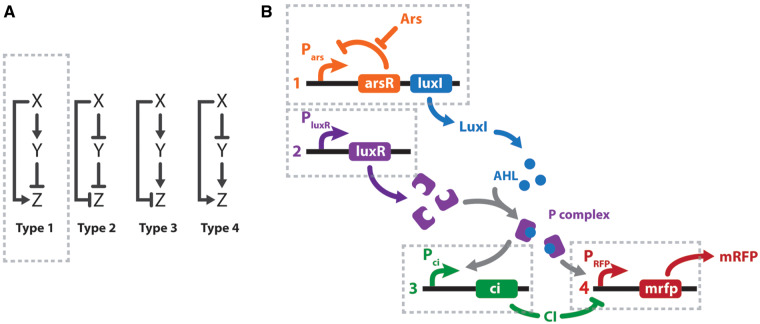
I-FFL schemes. (**A**) Canonical I-FFL circuits. Composing elements: input (*X*), auxiliary regulator (*Y*), and output (*Z*). (**B**) Design of a genetic type 1 I-FFL or I1-FFL circuit from BioBricks. Transcription units are numbered 1–4 under the regulation of *P_ars_*, *P_luxR_*, *P_ci_* and *P_hyb_* promoters, respectively. Arsenic (Ars) and mRFP are the input and output signals of the system. To exploit the modular design, the sensor (transcription unit 1) and effector (transcription units 2–4) modules could be located in two different bacteria. Activation is represented by normal arrows and repression by barred arrows.

Feed-forward and feed-back loops are the most usual motifs found among biological networks (8). The canonical FFLs contain three elements: input (*X*), auxiliary regulator (*Y*) and output (*Z*). There are eight possible combinations of these three elements that give rise to ‘coherent’ and ‘incoherent’ loops. A scheme for the four possible different I-FFLs is depicted in Figure 1A. In all cases, the I-FFLs display contrary actions (i.e. stimulation and inhibition) on the output *Z*, exerted by the input *X* directly and indirectly via the auxiliary regulator *Y* (Figure 1A). In the particular case of type 1 incoherent feed-forward loops (I1-FFL), *X* stimulates *Z* directly, also stimulates *Y*, while the latter inhibits *Z*. Interestingly, I1-FFL comprise about one-third of the possible FFLs identified in the transcriptional circuits in *E. coli* and *Saccharomyces cerevisiae* (9). I1-FFL function as pulse-like generators of *Z* in response to continuous *X* stimuli and also act as response accelerators, meaning that *Z* may reach steady states more rapidly than in non-regulated circuits.

Using ordinary differential equations (ODEs), we generated a mathematical model consisting of an I1-FFL system that senses arsenite and produces a reporter protein as the output. Our model behaved as expected, showing a peak for the reporter protein production after arsenite induction that correlated to its input level. Moreover, subcircuits of the designed circuit were constructed and validated. This work underlines the importance of an integrated experimental and theoretical approach in synthetic biology, as well as the power and variety of the available biological parts in the Parts Registry (http://parts.igem.org/Main_Page), which can be reused in sophisticated system designs such as an I-FFL. Finally, we present the deep impact this project had on our careers both relative to the project itself and the way we pursued it further following the competition.

## 2. Materials and methods

### 2.1 System modelling

For modeling the system schemed in Figure 1B, we implemented ODEs, as a standard approach to model gene expression. Parameters are listed in Table 1. We estimated some of the parameters from *ad hoc* calculations, while others were taken from the iGEM Aberdeen Team 2009 (http://2009.igem.org/Team: Aberdeen_Scotland/parameters), as indicated in Table 1. To describe gene transcription by a transcription factor, we employed Hill input functions. Our model considers transcription and translation separately to analyze independently protein and messenger RNA (mRNA) concentrations. The equations were solved using a Runge–Kutta routine in C (code provided in Supplementary Data). Data were plotted using GraphPad Prism 5 software.

**Table 1.** Arsenic and related parameters used in this work

Parameter	Value	Units	Reference
*ΩP_ars_*	10	# plasmids	Aberdeen Team 2009
*ΩP_luxR_*	10	# plasmids	Aberdeen Team 2009
*ΩP_ci_*	10	# plasmids	Aberdeen Team 2009
*ΩP_hyb_*	10	# plasmids	Aberdeen Team 2009
*βP_ars_*	0.4	POPS	Estimated, this work
*βP_luxR_*	0.02	POPS	Estimated, this work
*βP_ci_*	0.4	POPS	Estimated, this work
*βP_hyb_*	0.4	POPS	Estimated, this work
*α_ArsR_*	0.0001	1/s	Estimated, this work
*α_LuxI_*	0.00288	1/s	Aberdeen Team 2009
*α_LuxR_*	0.0002	1/s	Aberdeen Team 2009
*α_P_*	0.0002	1/s	Aberdeen Team 2009
*α_CI_*	0.00288	1/s	Aberdeen Team 2009
*α_AHL_*	0.00016667	1/s	Aberdeen Team 2009
*α_mRFP_*	none	1/s	Estimated, this work
*k_P_*	0.00001	1/s	Aberdeen Team 2009
*k_−P_*	0.0033	1/s	Aberdeen Team 2009
*K_ci_*	7000	# molecules	Aberdeen Team 2009
*K_hyba_*	700	# molecules	Estimated, this work
*K_hybi_*	300	# molecules	Estimated, this work
*n_ars_*	1		Estimated, this work
*n_ArsR_*	1		Estimated, this work
*n_P1_*	2		Estimated, this work
*n_P2_*	2		Estimated, this work
*n_ci_*	2		Aberdeen Team 2009
*γ_protein_*	0.1	1/s	Aberdeen Team 2009
*θ_AHL_*	0.4	1/s	Aberdeen Team 2009
*μ*	0.45	1/s	Aberdeen Team 2009

### 2.2 Family of gene input functions (Hill input functions)

Hill function for activator:

$$f\left(\left[X\right]\right)=\frac{\beta {\left[X\right]}^{n}}{K^{n}+{\left[X\right]}^{n}}.$$

Hill function for repressor:

$$f\left(\left[X\right]\right)=\frac{\beta }{1+{\left(\frac{\left[X\right]}{K}\right)}^{n}}.$$

Hill function for repressor stimulated by inducer:

$$f\left(\left[X\right]\right)=\frac{\beta }{1+{\left(\frac{\left[X\right]}{K\left(1+\frac{\left[I\right]}{K_{I}}\right)}\right)}^{n}},$$

where *β* is the maximal expression level of the promoter, [*X*] is the concentration of protein *X*, *K* is the activation coefficient and *n* is the Hill coefficient. It defines the amount of *X* needed to significantly activate or repress a promoter. [*I*] is the concentration of the molecule that inhibits the repressor [*X*] where *K*_I_ is I inhibition constant to inhibit *X*.

### 2.3 Equations for mRNA transcription

Our system consists of four promoters, and models four different mRNAs, each one with different transcription rates depending on the promoter strength. Each type of mRNA was labeled using the names shown next to the promoter in Figure 1B. By linking one promoter with its mRNA products, we are assuming no difference in mRNA concentrations for different genes under the same promoter. This assumption is acceptable considering the lengths of our constructs.

Each equation describes the rate of transcription including a production term according to Hill functions competing against a degradation (*α*) and a dilution term. The dilution term was calculated considering one cell division every 30 min. The *Ω* parameter accounts for the number of plasmids.

[mRNA *P_ars_*]—repressed by ArsR which is inhibited by arsenic:

ddt[mRNAPars]=ΩParsβPars1+([ArsR]KArsR(1+[Ars]Kars)n ars)narsR−(αmRNA+ln (2)1800)[mRNAPars].

[mRNA *P_luxR_*]—constitutive promoter:

$$\frac{d}{dt}\left[{\text{mRNA}}_{P_{luxR}}\right]=\Omega_{P_{luxR}}\beta_{P_{luxR}}-\left(\alpha_{\text{mRNA}}+\frac{\ln\;\left(2\right)}{1800}\right)\left[{\text{mRNA}}_{P_{luxR}}\right].$$

[mRNA *P_ci_*]—activated by P complex:

$$\frac{d}{dt}\left[{\text{mRNA}}_{P_{ci}}\right]=\Omega_{P_{ci}}\beta_{P_{ci}}\frac{{\left[P\right]}^{n_{P1}}}{{\left[P\right]}^{n_{P1}}+K_{\text{CI}}^{n_{P1}}}-\left(\alpha_{\text{mRNA}}+\frac{\ln\;\left(2\right)}{1800}\right)\left[{\text{mRNA}}_{P_{ci}}\right].$$

[mRNA *P_hyb_*]—activated by P complex, repressed by lambda CI:

$$\frac{d}{dt}\left[{\text{mRNA}}_{P_{hyb}}\right]=\Omega_{P_{hyb}}\beta_{P_{hyb}}\frac{{\left[P\right]}^{n_{P2}}}{{\left[P\right]}^{n_{P2}}+K_{hyb}^{n_{P2}}}\frac{1}{1+{\left(\frac{\left[\text{CI}\right]}{K_{\text{CI}}}\right)}^{n_{CI}}}-\left(\alpha_{\text{mRNA}}+\frac{\ln\;\left(2\right)}{1800}\right)\left[{\text{mRNA}}_{P_{hyb}}\right].$$

We assume no competition between activation and repression because the binding sites of P complex and lambda CI are not physically entangled.

### 2.4 Equations for protein translation

Protein translation rate is proportional to the corresponding mRNA concentration, which competes against the degradation and dilution term calculated considering one cell division every 30 min.

- *γ* is the translation coefficient and is the same for all proteins.
- P is a complex of acyl–homoserine–lactone (AHL) and LuxR. Its degradation rate is the same as the one for LuxR, because the latter has a faster degradation rate than AHL. Upon P degradation, AHL remains.
- *k_P_* and *k*_*−*__*P*_ are the association and dissociation coefficients, respectively, of AHL and LuxR to form complex P.
- AHL diffuses through the cell membrane at a rate *θ*.
- *μ* is the coefficient of enzymatic production of AHL.

[ArsR]—represses *P_ars_*:

$$\frac{d}{dt}\left[ArsR\right]=\gamma_{\text{protein}}\left[mRNA_{P_{ars}}\right]-\left(\alpha_{arsR}+\frac{\ln\;\left(2\right)}{1800}\right)\left[ArsR\right].$$

[LuxI]— triggers AHL production:

$$\frac{d}{dt}\left[LuxI\right]=\gamma_{\text{protein}}\left[{\text{mRNA}}_{P_{ars}}\right]-\left(\alpha_{luxI}+\frac{\ln\;\left(2\right)}{1800}\right)\left[LuxI\right].$$

[LuxR]— forms complex protein P with AHL:

$$\frac{d}{dt}\left[LuxR\right]=\gamma_{\text{protein}}\left[{\text{mRNA}}_{P_{luxR}}\right]+k_{-P}\left[P\right]-k_{P}\left[AHL\right]\left[LuxR\right]-\left(\alpha_{luxR}+\frac{\ln\;\left(2\right)}{1800}\right)\left[LuxR\right].$$

[AHL]—forms complex protein P with LuxR:

$$\frac{d}{dt}\left[AHL\right]=\text{µ}\left[LuxI\right]+\left(k_{-P}+\alpha_{LuxR}\right)\left[P\right]-k_{P}\left[AHL\right]\left[LuxR\right]-\theta_{AHL}\left[AHL\right]-\left(\alpha_{AHL}+\frac{\ln\;\left(2\right)}{1800}\right)\left[AHL\right].$$

[P]—complex protein AHL + LuxR. Activates *P_ci_* and *P_hyb_*:

$$\frac{d}{dt}\left[P\right]=-k_{-P}\left[P\right]+k_{P}\left[AHL\right]\left[LuxR\right]-\left(\alpha_{P}+\frac{\ln\;\left(2\right)}{1800}\right)\left[P\right].$$

[CI]—inhibits *P_hyb_*:

$$\frac{d}{dt}\left[CI\right]=\gamma_{\text{protein}}\left[mRNA_{P_{ci}}\right]-\left(\alpha_{CI}+\frac{\ln\;\left(2\right)}{1800}\right)\left[CI\right].$$

[RFP]—colored pigment:

$$\frac{d}{dt}\left[RFP\right]=\gamma_{\text{protein}}\left[{\text{mRNA}}_{P_{hyb}}\right]-\left(\alpha_{RFP}+\frac{\ln\;\left(2\right)}{1800}\right)\left[RFP\right].$$

### 2.5 Design, construction and characterization of iGEM parts

All procedures to assemble new BioBricks from parts included in the 2013 iGEM distribution were performed according to the protocols reported at the Registry of Standard Biological Parts (http://parts.igem.org/Main_Page) and other well-described protocols (10). Resulting plasmids were characterized cloned in *E. coli* DH5α. Arsenite concentration curves were prepared from a sodium arsenite stock solution (5000 ppb) in liquid Luria-Broth medium (LB). Bacterial starter cultures were prepared inoculating a fresh colony grown on LB agar plates with the corresponding antibiotic in 2 ml of liquid LB, incubated at 30 °C overnight (16 h) under agitation (200 rpm). Then, 1 ml of the starter cultures was used to inoculate 10 ml of LB liquid medium and incubated at 30 °C under agitation (200 rpm) up to an optical density at 600 nm (OD600) of 0.5–0.6. Assays were performed by inoculating 25 μl of these cultures into 5 ml of LB liquid medium at different arsenite concentrations, incubating for 16 h at 30 °C, under agitation (200 rpm). After the incubation period, OD600 was recorded, and fluorescence was measured at the corresponding excitation–emission wavelengths (GFP: Exc. 475 nm, Em. 515 nm; mRFP: Exc. 585 nm, Em. 608 nm) (AMINCO-Bowman Series 2, Thermo Electron Corporation). The AHL detection assay was performed as previously described (11). Briefly, a layer of 0.5% (w/v) LB agar mixed with a *Chromobacterium violaceum* CV026 fresh culture was poured on top of a 1.5% (w/v) sterile LB agar plates. Holes of 100 μl were punched on the top agar layer and loaded with 60 μl of each sample culture. The top-agar plates were incubated overnight at 30 °C without agitation. Then, the production of violacein was evaluated and photographed for each well. *Rhizobium leguminosarum* A34 was used as a positive control (12).

## 3. Results

Our final goal was to develop a cheap and easy-to-read way of measuring arsenic concentration that would warn domestic users if it is above the allowed limits. We decided to design a circuit with a color development as the output, so it could be visible by the naked eye. This output should also scale with contaminant concentration and its development should be time independent. To achieve the latter, we designed a genetic circuit based on an I-FFL scheme. It was expected to generate a signal pulse dependent on the arsenic levels in the sample and making the output independent of the elapsed time once the peak was reached (13). We chose mRFP expression for color development as it was the only protein in the 2013 BioBricks distribution kit that could be easily detected by naked eye in our tests.

When choosing the network motif we took different things into account. Firstly, it was mandatory for us to assure a positive correlation between the arsenic concentration (input) and the visible output (i.e. mRFP concentration). Secondly, as mRFP is a highly stable protein, the circuit should stop its production at a certain point to avoid saturation. Finally, the system should be robust and reliable. Therefore, the I1-FFL seemed a very well-suited circuit. Inspired by a biological network previously published by Basu *et al.* (14) and by the availability of iGEM BioBrick parts, we designed our arsenic sensing circuit harboring an I1-FFL depicted in Figure 1B. To assess a modular design, the sensor and the reporter circuits were conceived to be housed in different bacteria, allowing detection of other contaminants with minimal genetic modifications. Inside the sensor bacteria, arsenic activates the transcription of AHL synthase (LuxI) by inhibiting the arsenic repressor (ArsR) (Figure 1B, transcription unit 1). AHL diffuses into the medium and into the reporter bacteria, which have an AHL receptor (LuxR) (Figure 1B, transcription unit 2). AHL binds to LuxR, generating the P complex that upregulates transcription of two genes: the mRFP reporter and the negative regulator CI (Figure 1B, transcription units 3 and 4). As the mRFP gene is under the control of a hybrid promoter, its transcription is activated by the P complex and repressed by CI. Thus, besides generating a pulse signal, an I1-FFL in this design helped to cope with amplification and saturation effects generated by LuxI and mRFP, respectively.

### 3.1 Incoherent feed-forward model and system response upon arsenic addition

To qualitatively understand the general behavior of our system and its feasibility, we used ODEs to generate a mathematical model of the system. Most of the parameter values employed were taken from Aberdeen iGEM Team 2009 (http://2009.igem.org/Team: Aberdeen_Scotland/parameters) and are listed in Table 1 (see Materials and methods). Because the coefficients of the arsenic promoter components were not available in the literature, we estimated them arbitrarily based on similar genetic components from the same work. Arsenic levels were simulated as they are found in contaminated water (>50 ppb).

**Figure 2.**
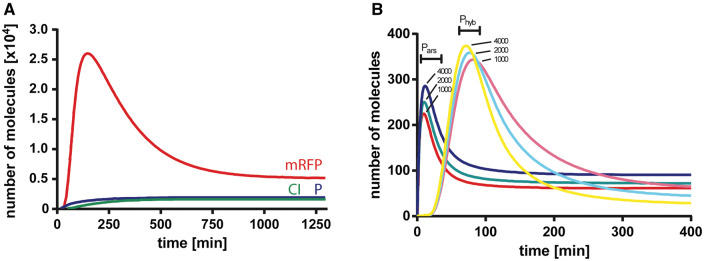
System evolution after arsenic stimuli. (**A**) Red fluorescent protein (mRFP, red), CI repressor (green) and P complex (blue) intracellular protein number were simulated immediately after arsenic was added to the system at *t* = 0. (**B**). Dose–response variation of mRNA levels after induction with different arsenic levels. Messenger RNA levels from *P_ars_* (red, green and blue) and *P_hyb_* (magenta, cyan and yellow) promoters are simulated after the addition of 1000, 2000 and 4000 arsenite molecules at *t* = 0. (B) depicts how the mRNA levels responded in a dose-dependent manner and in a relatively time-independent fashion upon the addition of arsenic. The mRNAs derived from *P_ars_* and *P_rfp_* were induced in the presence of different concentrations of arsenic. The *P_ars_* mRNA pulse, observed at *t* ∼ 10 min, is, in fact a damped oscillation product of the smooth Hill function that represents the repression of ArsR. *P_rfp_* mRNA levels are in very good agreement to the reporter gene from Basu *et al.* (14), where a similar biological system was designed.

To analyze the response of the system after the addition of arsenic, we simulated the concentration over time of mRFP, CI and the P complex. Figure 2A shows the evolution of individual components after the addition of arsenite at the beginning of the simulation, where a pulse of the reporter mRFP is observed after the production of the P complex. The mRFP decay is due to the generation of CI repressor, which halts transcription of the reporter, and dilution and degradation effects.

### 3.2 Experimental validation

**Figure 3.**
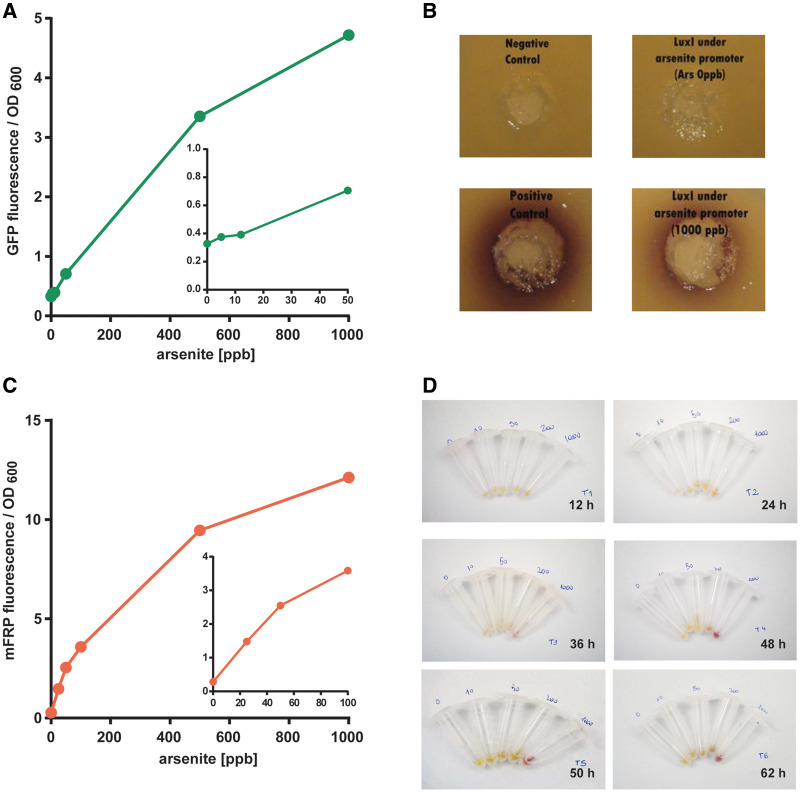
Characterization of constructed iGEM parts. (**A**) Fluorescence measurements from green fluorescent protein production under the regulation of *P_ars_* (Bba_K1106004) after 16 h and stimulated with sodium arsenite (0–1000 ppb). Data are expressed as fluorescence units relative to the culture OD600. Excitation: 475 nm, emission: 515 nm. The inset shows arsenite levels below 100 ppb. (**B**) AHL determinations in *C. violaceum* top agar wells inoculated with *E. coli* cultures. The production of AHL in the culture is evidenced by the violet ring developed. Cultures harboring LuxI under the regulation of *P_ars_* (Bba_K1106008) were incubated with 0 or 1000 ppb arsenite for 16 h. Negative control: *E coli* culture carrying a pSB1C3 empty plasmid. Positive control: *R. leguminosarum* culture. (**C**) Fluorescence measurements from mRFP production under the regulation of *P_ars_* (Bba_K1106003) after 16 h and stimulated with 25, 50, 100, 200 or 1000 ppb arsenite. Data are expressed as fluorescence units relative to the culture OD600. Excitation: 585 nm, emission: 608 nm. The inset above shows arsenite levels below 100 ppb. (**D**) Pellets from cultures harboring mRFP under the induction of *P_ars_* after incubation with 0, 10, 50, 200 or 1000 ppb arsenite. Aliquots of 1 ml were harvested and centrifuged after 12, 24, 36, 48, 50 or 62 h.

One of the main aims of our team facing the 2013 iGEM competition was to use exclusively the available BioBrick parts sent in the distribution kit or its combinations thereof. Therefore, we designed and constructed 10 new parts from the combination of previous parts (Table 2). To validate our theoretical model, we started assessing the behavior of each part. For this purpose, *E. coli* DH5α harboring a plasmid that encodes GFP expression under the regulation of *P_ars_* (Bba_K1106004) was cultured with increasing arsenite concentrations. The fluorescent signal was detectable using arsenite concentrations below 50 ppb (Figure 3A), thus within the required range. Then, we characterized the function of LuxI under the arsenite inducible promoter (Bba_K1106008). To do this, we used the bacterium *Chromobacterium violaceum* CV026 as an AHL detection tool, which produces the purple color pigment violacein in the presence of AHL (11). The strain CV026 is impaired in AHL biosynthesis, therefore the color violacein signal arises only from external AHL signal molecules. We inoculated wells in *C. violaceum* top agar plates with different bacterial cultures (Figure 3B). Violacein production can be observed upon inoculation with *E. coli* harboring the Bba_K1106008 part and stimulated with arsenite at 1000 ppb. As expected, there was no color production upon inoculation with unstimulated (0 ppb arsenite) Bba_K110600- harboring *E. coli* or with stimulated bacteria (1000 ppb) lacking LuxI. As a positive control, we used a *R. leguminosarum* culture (Figure 3A), which naturally produces AHL (12). In conclusion, the part Bba_K1106008 works as expected and the first part of our I1-FFL system was validated.

**Table 2.** New BioBricks submitted in 2013 by our team to the Parts Registry

Registry code	Type	Sequence description	Length
Bba_K1106000	Composite	mRFP generator under hybrid promoter: AHL-LuxR activated, P22 C2 repressor	938 bp
Bba_K1106001	Composite	P22 c2 generator under PLL promoter	970 bp
Bba_K1106002	Composite	psp3 generator under lux pR promoter	437 bp
Bba_K1106003	Reporter	mRFP generator under *ars* promoter	1390 bp
Bba_K1106004	Reporter	GFP generator under *ars* promoter	1404 bp
Bba_K1106005	Composite	LuxR generator under a constitutive promoter	982 bp
Bba_K1106006	Reporter	Pulse generator part 3	1928 bp
Bba_K1106007	Composite	Pulse generator part 2 (not sent)	1415 bp
Bba_K1106008	Composite	Pulse generator part 1	1327 bp
Bba_K1106009	Generator	LuxR generator with a strong RBS	939 bp

As we intended to use a reporter protein that could be visible to the naked eye, in the next stage, we constructed and characterized a part consisting of mRFP under *P_ars_* regulation (Bba_K1106003) in *E. coli* DH5α. First, we measured mRFP fluorescence upon arsenite induction using a fluorimeter. The mRFP fluorescence was produced in a dose-response manner in the range tested (0–1000 ppb), with a clear signal starting at 25 ppb (Figure 3C). Then, we performed a study of mRFP color intensity development over time to assess detection by the naked eye and determine the saturation time. For this purpose, cultures were grown with different arsenite concentration (0, 10, 50, 200 and 1000 ppb), then 1 ml aliquots were harvested after 12, 24, 48, 50 and 62 h and pelleted by centrifugation. Pictures of the pellets can be observed in Figure 3D. After 24 h of high arsenite concentration induction (1000 ppb), the color production is clearly distinguishable from the unstimulated control. However, it took 48 h to observe a clear color change when inducing with lower concentrations (∼50 ppb). Unfortunately, the differences between 0 and 10 ppb arsenite cultures were difficult to discriminate (Figure 3B). It can also be noticed that the mRFP production seems to reach a saturation limit after 48 h in the 1000 ppb arsenite treatment.

## 4. Discussion

Our goal was to engineer an arsenic biosensor based on standard biological parts. We chose an I1-FFL to obtain an output pulse independent of elapsed time. We were able to build a computational model of the system, which presented good accordance with experiments and with a previous system with similar characteristics (14). We could obtain a signal peak that was dose dependent upon arsenic addition. However, a drawback of our model can be observed in Figure 2B, where at later times, higher input signals produced lower mRNA level. This behavior may be caused by very strong CI repression.

During the course of this work, we realized that generating a mathematical model from scratch is a fairly difficult task and parameters mainly based on data presented by other iGEM groups. It would be substantially beneficial for future teams and synthetic biology projects if models, parameters and other contributed tools were somehow gathered in a searchable database and normalized using a reference standard (e.g. POPS) (15).

We have implemented our design for an *E. coli* chassis exclusively based on standard parts distributed in iGEM kits for the standard assembly method. This work is an example of the power and variety of the available biological parts, which can be reused to design systems as complicated as an I-FFL. We further contributed components to the iGEM Registry of Standard Biological Parts that have been used by other teams. We have designed and constructed 10 new parts (Table 2) needed to both validate the components of our system and, ultimately, produce the complete biosensor modeled *in silico*. We characterized the *P_ars_* promoter regulating three different genes: two reporter proteins, namely GFP and mRFP by measurement of fluorescence and color production, and LuxI by measuring the production of AHL revealed by a *C. violaceum* biosensor strain.

Our results show that, although the I1-FFL system achieved a promising performance *in silico*, it would not fulfill the need to reach a clearly appreciable signal to the naked eye on water samples close to the arsenic concentration limit set by the WHO (which will drop from 50 ppb to 10 ppb in 2017: http://www.who.int/mediacentre/factsheets/fs372/en/). Moreover, it would require an incubation time impractical to use in an arsenic field kit. Taking these results into consideration, we decided not to pursue the experimental validation of the whole I1-FFL circuit but rather focus on designing a good internal reference. Nevertheless, all the parts needed to build our modeled arsenic biosensor were constructed, sent and registered in the iGEM Parts Registry (Table 2).

To achieve our main goal, we are developing an arsenic biosensor that allows a non-trained user to decide whether their water source contains a toxic arsenic concentration. We opted for using a simpler genetic system, complemented by the addition of different arsenic concentrations to independent wells as internal color development reference. We are evaluating other improvements to reduce the incubation time and lower the detection limit. To this end, we are studying different molecular designs and testing enzymatic reporter proteins such as LacZ (4). Other improvements include the addition of an extra ArsR binding region upstream of the reporter gene to block the leaky transcription of the reporter under the control of *P_ars_*, increasing the signal-to-noise ratio (16); or the introduction of an RBS sequence in the same region to accelerate the transduction time of the reporter, as reported by Wackwitz *et al.* (17). With all these improvements enhancing the genetic circuit and the assistance of industrial designers, we are developing a case that could deliver the biosensor in a safe, cheap and easy-to-use manner to be used by the general population (http://sensar.com.ar/).

## 5. Educational impact

The aim of this section is to share the enthusiasm that worked as an engine and driving force for an interdisciplinary group of students and teachers from the University of Buenos Aires to build an arsenic biosensor based on synthetic biology. In only 2 years, the project evolved from the iGEM competition starting point to an applicable prototype which received a major innovation award (http://en.mincyt.gob.ar/news/winners-of-the-innovar-2014-were-announced-9588).

Everything started much before knowing about arsenic-contaminated waters or its impact in Argentina. As a consequence of the work performed by the 2012 Buenos Aires iGEM team (http://2012.igem.org/Team: Buenos_Aires) and its transforming experience, we realized that a successful iGEM project should continue beyond the Jamboree. Thus, we proposed to work on a project that could potentially impact on our society. We started by defining that the project would aim to contribute to warn vulnerable domestic users about arsenic contamination. Then, we designed a system that could work as a biosensor and modeled it. A genetic circuit was designed after discarding alternative models that probably would not work or those for which there were no biobricks available (http://2013.igem.org/Team: Buenos_Aires/_model). Then, we started to build basic modules based on standard parts and protocols distributed in the 2013 iGEM kit. While we were evaluating those basic modules for constructing the whole system, we contacted industrial designers to help us design a device that could ‘contain’ our bacterial-based biosensor. Initially, we expected to transmit them a couple of requirements to hold viable bacteria and then outsource that part of the project to them. Fortunately, what happened was quite different: they taught us that to do their job adequately they needed to understand what we were doing, how we were doing it, our motivations and who was going to be the user. Thus, they got involved in the development as peers. This represented a challenge and an opportunity for us to learn some rudiments about design, and for them, some of synthetic biology. Then, we started thinking together how to design the device to meet the desired requirements of being accessible to our target users and affordable to be produced industrially. This involved, for example, learning about materials, processes, usability and communication. Interacting with designers was highly enriching and prompted us to exit the four walls of the lab towards the vulnerable people exposed to arsenic, the users and the production systems.

The team had a horizontal organization, where all of us were peers working on identifying the problem, the ways to tackle it and how to execute the plan. We all debated about intellectual property, time management and financial issues. In terms of organization, both advisors and students chose different days of the week to work in small teams in the wet and dry laboratories. This required outstanding communication between the team members and full comprehension of the protocols carried out every day. To achieve this, coordination meetings were implemented frequently as well as the use of a laboratory notebook. The horizontal approach of decision-making also presented us some difficult moments, apparently making the process longer than it would have been in a hierarchical organization. We think, however, that the creativity and empowerment that it promotes overcome the downsides. This introduction into project management suddenly unveiled the large gap between the curricula from scientific academic degrees and the skills that are necessary to carry on a successful technological project. Thus, we think that the educational process was, at least, of equal importance to the laboratory outcome. We all had to learn how to be self-organized and plan as a team, formally apply for access to a workspace to the university’s authorities, participate in ordering reagents in the lab, raise funds, overcome unexpected bottlenecks. Moreover, we had to study about relevant national and international regulations and we exposed the arsenic problems and our project in conventional and social media. As a consequence, since 2016 we have been organizing a Latin American technological competition, named TECNOx (http://blogs.plos.org/synbio/2016/02/09/tecnox-a-latin-american-syn-bio-and-more-student-competition/; www.tecnox.org), that feeds from the most useful practices from the iGEM experience but that is adapted to the Latin American schedules, culture and resources. Furthermore, the aim is to promote a regional technological community, starting from early career students, that could tackle regional problems. We have already organized two successful TECNOx editions and the third is being organized in Chile for 2018. During these competitions, we have offered workshops on soft skills. Moreover, we have proposed the inclusion of these workshops to our Faculty’s curricula.

Financing was a very challenging issue. Getting the required amount of resources for materials and buying reagents as well as for travelling expenses to the jamborees was beyond any standard path we knew in our country because of the high amount (several thousand dollars), the type of project (not for basic nor translational science; not defined *a priori* but during the competition) and the team composition (not a standard laboratory with a principal investigator, but many PhDs and students from many places). The infrastructure and some consumables were provided by the University’s Biological Chemistry Department. Some research laboratories also contributed by donating some reagents. We initiated fund-raising actions that consisted of contacting private companies asking for donations and a crowdfunding campaign (https://social.idea.me/projects/10897/sensar-water-biosensors). Finally, we asked for support from the Ministry of Science and Technology, which underwrote the remainder of the budget so that we could travel and represent our country in an international competition. To open the doors to funders, collaborators, non-governmental organizations (NGOs) and to the general public, we had to rely on all the outreach skills we had learned until then as well as to communicate our project in a smooth and appealing way. We offered talks to high schools, several press notes (in radio, TV and written media) and had the opportunity to give TEDx (http://www.tedxriodelaplata.org/videos/biologia-sintetica; https://www.youtube.com/watch? v=8I5mqniNOlg) and Campus Party talks, (http://campuse.ro/events/campus-party-argentina-2016/talk/biologia-sintetica-bio-tecnologia-para-tods-alejandro-nadra-cpar1/; http://campuse.ro/events/campus-party-ecuador-2015/talk/biologia-sintetica-bio-tecnologia-para-tods-alejandro-d-nadra-cpec5/) reaching a wide audience.

Finally, prizes should not be the goal for a project like this, but they are welcome, rewarding effort and providing opportunities to make the project visible. Besides a gold medal and best model prize obtained in iGEM 2013, our project participated in national innovation contests, receiving an innovative product award at Innovar 2014 (http://en.mincyt.gob.ar/news/winners-of-the-innovar-2014-were-announced-9588), a mention in Alltec100k 2016 (a local innovation contest: https://www.funintec.org.ar/ganadores-alltec-2016/) and a prize in ‘Eureka desafío de ideas 2016’ (TV show: http://www.desafioeureka.com/).

Participating in the Buenos Aires iGEM team has been an enriching experience for both students and advisors. Most of us had never worked on an interdisciplinary project, and this was a unique opportunity. It was also our first time developing a product for the general public, which, in turn, led us to improve our science outreach and communication skills. Since the goal was to design a useful domestic device, we interacted with affected people, NGOs and Science and Health authorities. They perceived our proposal as a new and potentially useful tool to tackle a widespread social problem and were willing to contribute to its testing and development. Furthermore, a horizontal collaboration between early students and established researchers led to the combination of enthusiasm and experience into a stimulating balance. Particularly for students, this was a rare opportunity to choose a project, work on it and arrive at a conclusion. It required many skills that are not usually taught in the classroom such as project development, application writing, science communication and fund-raising. Also, it pushed all the members to learn content outside our major curricula: chemistry and molecular biology for physicists and computer scientist and modeling and simulation for biologists and chemists. Having combined two or more academic disciplines not only allowed us to go beyond our expectations but also taught us that in the field of synthetic biology interdisciplinary collaboration is indispensable to create novel biological applications. As a result, students and instructors widened their views and reshaped their careers in significant ways. After the iGEM experience, most of the students completed their undergraduate thesis and are now doing a PhD on diverse areas, some of them related to synthetic biology such as systems biology, protein engineering, bioinformatics and bionanotechnology (see Table 3). One of the instructors, trained in informatics, is currently enrolled in a Master’s degree in bioengineering. For another instructor, a professor who came from the traditional academic career path, this experience opened a window toward technological development and innovative educational experiences such as TECNOx, where much of the experience we gained has been incorporated. In summary, the iGEM 2013 experience challenged our way of working and thinking and that changed all of us forever.

**Table 3:** Members of the iGEM Buenos Aires 2013 Team

Name	Role	Background	Impact of IGEM on his/her career and current position
Federico Barone	Student	Physics	MSc student in Physics
Francisco Dorr	Student	Computer science	MSc student in Computer Science
Luciano E. Marasco	Student	Biology	PhD student in epigenetics of neuromuscular disorder at IFIBYNE
Sebastian Mildiner	Student	Biology	MSc student in Methodology and Statistics for the Biomedical, Behavioral and Social Sciences at Utrecht University
Ines L. Patop	Student	Biology	Finished undergraduate thesis in Systems Biology under Alejandro Colman-Lerner’s direction
			PhD student Marie Curie Innovative Training Network in Molecular biology and Neurobiology impacts of circRNA
Santiago Sosa	Student	Biology	Finished undergraduate thesis in protein engineering under Hernán Bonomi’s direction
			PhD student in Nanobiotechnology at Leloir Institute-CONICET
Lucas G. Vattino	Student	Biology	PhD student in Neuroscience at INGEBI-CONICET
Federico A. Vignale	Student	Biology	Nanobiotechnology Internship under Patricio Craig’s direction
			PhD student in Microbiology at IQUIBICEN-CONICET
Edgar Altszyler	Advisor	Physics	PhD in Physics
Benjamin Basanta	Advisor	Biology	PhD student at University of Washington, Baker Lab
Nicolás Carlotto	Advisor	Biology	First experience in an interdisciplinary group. Acquired basic concepts of system biology and modeling
			PhD student in Plants Molecular Biology at University of Buenos Aires
Javier Gasulla	Advisor	Biochemistry	Postdoc fellow in Synthetic Biology at IQUIBICEN-CONICET
Manuel Giménez	Advisor	Computer science	MSc in Biomedical Engineering at Boston University
Alicia Grande	Advisor	Biology	Postdoc fellow in Systems Biology at IFIBYNE-CONICET
Nicolás Nieto Moreno	Advisor	Biology	PhD student in Molecular Biology at IFIBYNE-CONICET
Hernán R. Bonomi	Advisor	Biology	Incorporated protein engineering concepts to his research
			Advisor of Santiago Sosa for his licentiate thesis on protein-oligomeric state manipulation
			Researcher in Molecular Microbiology at Leloir Institute-CONICET
Alejandro D. Nadra	Instructor	Biology	Researcher at CONICET and professor at the school of sciences of the UBA
			Co-founder of TECNOx
			Involvement in technology development
			Applying innovations in biology courses
			Moved its research topics toward interdisciplinary approaches

## Supplementary data

Supplementary Data are available at SYNBIO Online.

## Supplementary Material

Supplementary Data
